# Double Charge Ordering States and Spin Ordering State Observed in a RFe_2_O_4_ System

**DOI:** 10.1038/srep06429

**Published:** 2014-09-19

**Authors:** Fei Sun, Rui Wang, C. Aku-Leh, H. X. Yang, Rui He, Jimin Zhao

**Affiliations:** 1 Beijing National Laboratory for Condensed Matter Physics and Institute of Physics, Chinese Academy of Sciences, Beijing 100190, China; 2 Department of Physics, University of Northern Iowa, Cedar Falls, Iowa 50614, USA; 3 ISciences, Ann Arbor, Michigan, 48103, USA

## Abstract

Charge, spin, and lattice degrees of orderings are of great interest in the layered quantum material RFe_2_O_4_ (R = Y, Er, Yb, Tm, and Lu) system. Recently many unique properties have been found using various experimental methods. However so far the nature of the two-dimensional (2D) charge ordering (CO) state is not clear and no observation of its fine structure in energy has been reported. Here we report unambiguous observation of double 2D CO states at relatively high temperature in a polycrystalline Er_0.1_Yb_0.9_Fe_2_O_4_ using Raman scattering. The energy gaps between the 3D and the double 2D states are 170 meV (41.2 THz) and 193 meV (46.6 THz), respectively. We also observed a spin ordering (SO) state at below 210 K with characteristic energy of 45 meV (10.7 THz). Our investigation experimentally identified new fine structures of quantum orders in the system, which also extends the capability of optical methods in investigating other layered quantum materials.

Unique properties based on various orderings in complex materials, especially the RFe_2_O_4_ system, have drawn great interest recently^1^,^2^,^3^,^4^,^5^,^6^,^7^,^8^,^9^,^10^,^11^,^12^,^13^,^14^,^15^,^16^,^17^,^18^,^19^,^20^,^21^. Experiments such as x-ray diffraction^1^,^2^, neutron scattering^4^,^13^, electronic transport measurement^8^, *in situ* cooling transmission electron microscopy^23^, optical spectroscopy^6^,^17^, *etc.* have been carried out and illustrated various types of orderings and properties such as charge ordering^5^,^9^, spin ordering^1^,^5^, giant magnetocapacitance^9^, giant room-temperature magnetodielectric response^10^, giant magnetic anisotropy^20^, *etc* in the system. However, there are very few reports on the detailed energy structure of the 2D CO states and optical methods have been less reported in identification of the spin ordering state. In this paper we show clear evidence of observing non-degenerate *double* 2D CO states and a ferrimagnetic SO state in an electronic ferroelectric material Er_0.1_Yb_0.9_Fe_2_O_4_ by using temperature-dependent Raman scattering. Furthermore, we have also observed two infrared-active phonon modes besides the full spectrum of Raman-active phonon modes in it. This is a material with which we had for the first time observed the Stark effect in a solid, which confirmed the existence of strong local field and provided a spectroscopic evidence of the ferroelectric nature of the material^7^.

The Er_0.1_Yb_0.9_Fe_2_O_4_ crystal belongs to the RFe_2_O_4_ type mixed-valence materials, which has been known exhibiting CO composing Fe^2+^ and Fe^3+^ ions on a geometrically frustrated triangular lattice. The bulk ferroelectric polarization thus formed (*i.e.* electronic ferroelectricity) arises from the 3D alternating arrangement of valence-charges, instead of the spatial displacement of cations as in the conventional ferroelectric materials^1^,^23^. Among the many investigation methods, Raman and infrared (IR) spectroscopy investigations have been carried out on LuFe_2_O_4_^6^,^16^,^17^,^18^, illustrating its structural, magnetic and charge ordering properties. In the IR investigation a transition between the 2D and the 3D CO was observed^17^. However, so far Raman and IR results have not provided any information on spin ordering in the RFe_2_O_4_ system, although neutron diffraction have been used to find the ferrimagnetic order in LuFe_2_O_4_^22^.

In this Article, a temperature-dependent Raman scattering study on the phase transition in polycrystalline Er_0.1_Yb_0.9_Fe_2_O_4_ has been carried out. Unlike available reports on Raman scattering and other optical spectroscopy of the RFe_2_O_4_ system, our experiment presents a full spectrum of the excitations and show five distinct new modes. Significantly we observed two non-degenerate 2D CO modes at the temperature range of 300 ~ 400 K. We directly obtained the energy gaps between these 2D double CO states and the 3D CO state. Also we have observed one mode attributed to the ferrimagnetic spin ordering at below 210 K. Furthermore two IR-active modes have also been identified due to the breakdown of inversion symmetry at the crystalline domain boundaries. Our investigation extends or demonstrates the Raman (and optical) investigation capability in identifying the phase transitions and various orderings in such complex quantum materials.

## Results

**Figure 1:**
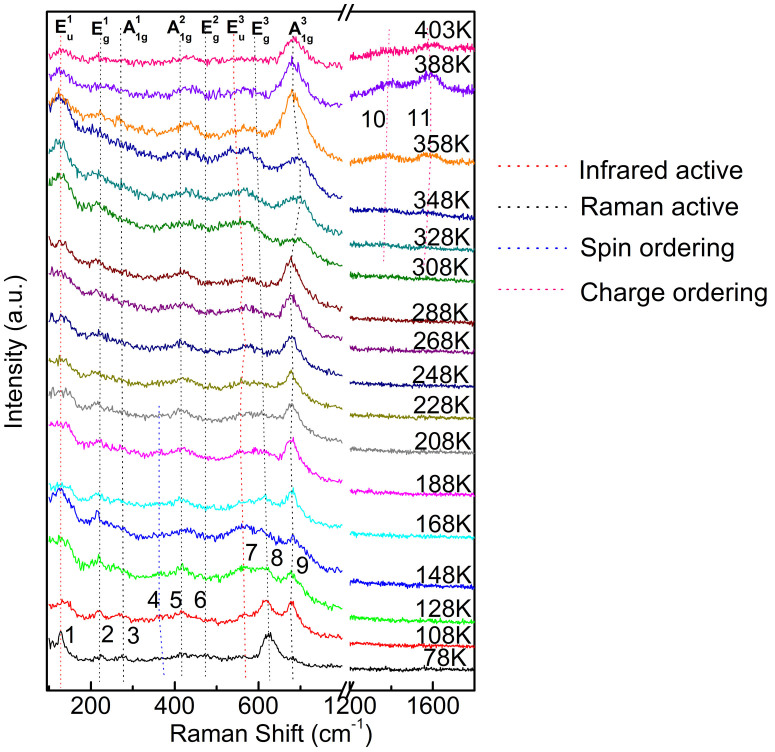
Raman spectra of Er_0.1_Yb_0.9_Fe_2_O_4_ at various temperatures. Raman peaks are numbered and highlighted by dashed lines.

**Figure 2:**
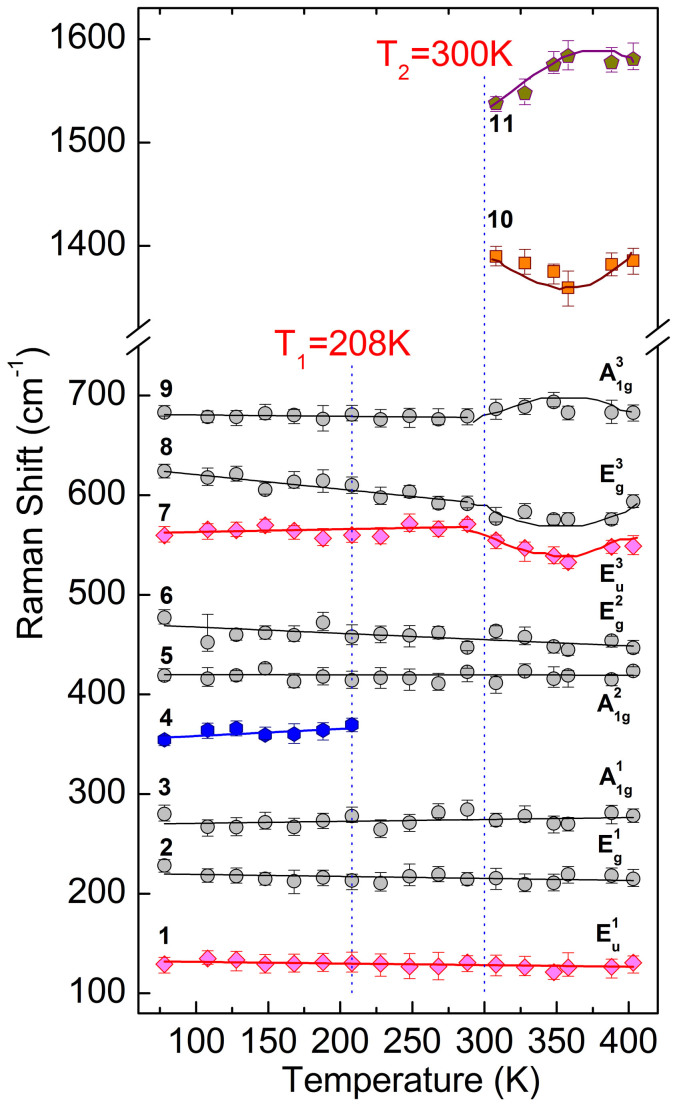
Frequency of the 11 Raman modes highlighted in Fig. 1 as a function of temperature. The vertical dashed lines highlight the temperatures at which phase transitions occur. The solid lines are guides to the eye.

**Figure 3:**
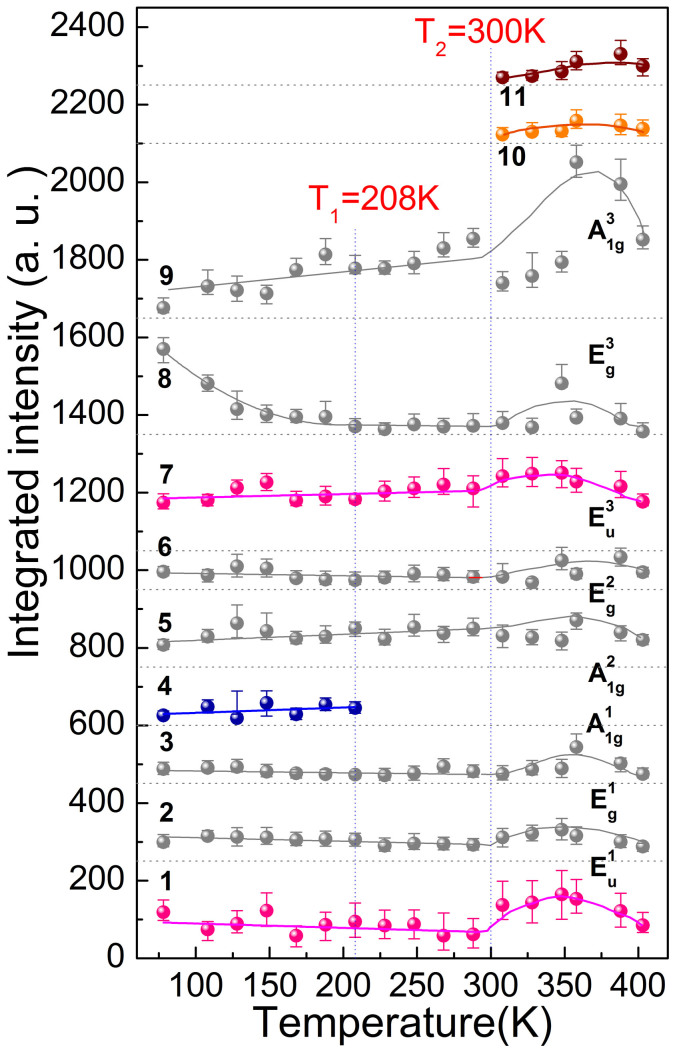
Integrated intensities of the 11 Raman modes (see Fig. 1) as a function of temperature. The vertical dashed lines highlight the temperatures at which phase transitions occur. The intensity for each mode is shifted vertically for clarity. The solid curves are guides to the eye. The horizontal dotted lines below each solid curve represent the base line of intensity without offsets.

Variable-temperature Raman spectra of our polycrystalline Er_0.1_Yb_0.9_Fe_2_O_4_ are shown in Fig. 1. The temperature dependences of the frequencies and intensities of all the observed modes are shown in Figs. 2 and 3, respectively. We observed 11 modes altogether, of which the information is summarized in Table 1.

Significantly, two high frequency modes (numbered 10 and 11 in Figs. 1–3) begin to emerge when the temperature *T* is raised to higher than 300 K. These two Raman peaks cannot be assigned to higher-order Raman or IR modes since they appear only when *T* is higher than 300 K. Various diffraction techniques and theoretical analysis have been applied to the LuFe_2_O_4_ systems, which suggested a 3D CO to 2D CO transition that occurs at a similar temperature when temperature is raised^5^,^24^. Here we assign these two high frequency modes to the 2D CO states in the Er_0.1_Yb_0.9_Fe_2_O_4_ crystal. The energy scale of these two modes is at the same order as those observed in other RFe_2_O_4_ systems using other methods^3^,^25^ (see below discussion). In light of the relatively high frequencies of these two modes we speculate that these two modes are due to in-plane interactions, which is relatively stronger due to closer distance between atoms and consistent with the 2D nature of the CO state.

As expected (see Discussions) all six Raman-active modes (with subscripts *g*) are observed (highlighted by black dashed lines in Fig. 1 and gray points in Fig. 2). Among them, the three Raman modes of A_1g_ symmetry are due to the out-of-plane vibrations (where atoms move perpendicularly to the Yb-O, Er-O, and Fe-O planes, respectively), and the three *E*_g_ symmetry Raman modes corresponds to the in-plane vibrations^6^. A pair of modes (modes 1 and 7) exist in the entire range of temperature (highlighted by red dashed lines in Fig. 1 and red points in Fig. 2), whose frequencies are close to those of the E_u_^1^ and E_u_^3^ vibrations in the RFe_2_O_4_ type crystals^6^. We assign these two modes to the IR-active modes with E_u_^1^ and E_u_^3^ symmetries, respectively. Usually the IR active modes are not expected in Raman measurements for this material. The observation of the E_u_^1^ and E_u_^3^ symmetry modes can be well understood based on the polycrystalline nature of the sample. The rich boundaries and vacancies in the sample break down the inversion symmetry of the crystal at the surfaces and the phonon parity selection rule is largely relaxed in our polycrystalline sample.

In Fig. 2 we can see that the frequencies of three Raman-active modes E_g_^1^, A_1g_^1^, A_1g_^2^ and the lowest energy IR-active mode E_u_^1^ do not show much variation in the entire temperature range. The E_g_^2^ mode softens from 477 cm^−1^ to 447 cm^−1^ when *T* changes from 78 K to 403 K. The frequencies of the three higher energy vibrational modes E_u_^3^, E_g_^3^, and A_1g_^3^ show non-monotonic dependence on *T*. When *T* varies from 78 K to 300 K, the E_u_^3^ vibration hardens slightly, whereas the E_g_^3^ vibration redshifts from 626 cm^−1^ to 593 cm^−1^. The A_1g_^3^ vibrational frequency remains unchanged in this temperature range. For *T* > 300 K, the E_u_^3^ and E_g_^3^ vibrational frequencies reach a minimum at about 350 K, and the A_1g_^3^ frequency reaches a maximum at *T* ~ 350 K. The spin-ordered mode 4 blueshifts slightly when *T* increases from 78 K to ~ 210 K, and it disappears for *T* higher than 210 K. The two highest frequency modes (numbered 10 and 11) only appear when *T* is higher than 300 K. The frequency of mode 10 has a minimum value at *T* ~ 350 K, whereas the frequency of mode 11 has a maximum value at *T* ~ 350 K.

In Fig. 3 we show that the intensities of modes 1–3 and 5–7 do not display significant changes for *T* < 300 K. The intensity of mode 8 decreases when *T* changes from 78 K to 210 K. Between 210 K and 300 K, the intensity of mode 8 does not change. Specifically, at around 150 K, the mode intensity decreases and a transfer of oscillator strength occurs the surrounding background gains weight. For mode number 9, its intensity changes when *T* changes from 78 K to 300 K. All modes (modes 1 to 11 except mode 4) show their strongest intensities at *T* ~ 350 K. The intensity of mode 4 which we attribute to spin ordering does not show noticeable changes for the temperature range of 78 K ~ 210 K.

## Discussion

### Charge ordering phase transition

**Figure 4:**
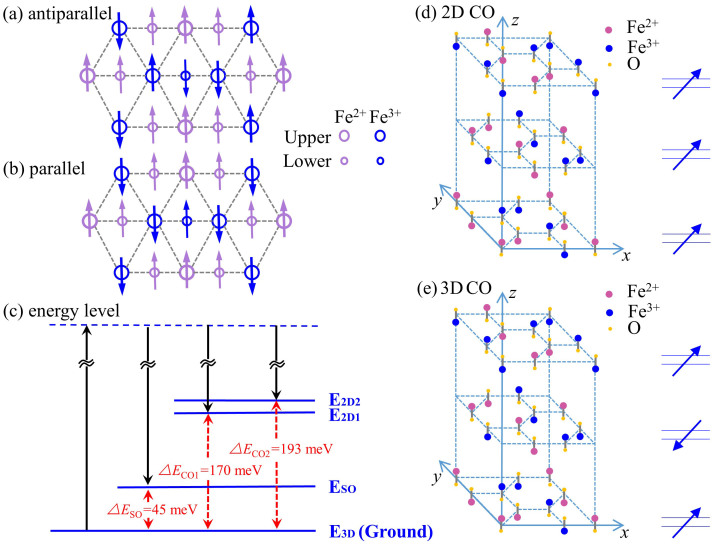
Fine structure in energy for the orderings. The anti-parallel (a) and parallel (b) spin orderings in the material [Ref. 27]. (c) The schematic energy level for the double 2D COs, the SO, and the 3D CO with the energy gap values marked explicitly. Note the 3D CO level is very close to the ground state. Schematic lattice structures of the (d) 2D CO states and the (e) 3D CO state, where the polarization is marked by arrows explicitly on the right panel [Ref. 2].

Since Fe ion has an average valence of 2.5+, equal numbers of Fe^2+^ and Fe^3+^ ions co-exist in a lattice unit cell. Compared to the average valence of 2.5+ for the Fe ions, the Fe^2+^ and Fe^3+^ ions are practically equivalent to possessing a 0.5− and 0.5+ valence, respectively. In the triangular lattice charge frustration between the 0.5- and 0.5+ ions is thus inevitable, which leads to various forms of charge orders. Most noticeably charge order phase transition has been confirmed by various scattering experiments, such as X-ray scattering, neutron scattering, electron diffraction, *etc*^2^,^3^,^22^,^26^,^27^. For example, LuFe_2_O_4_ exhibits a phase transition at 330 K, where a 2D CO emerges and replaces the low temperature 3D CO and persists up to 500 K^2^,^28^. It is speculated that the Fe^2+^ and Fe^3+^ ions arranges themselves such that a 2D superstructure is formed within each individual W layer, largely owing to the stronger intralayer correlation^24^, as well as the interlayer correlation between W layers^29^,^30^. Here we attribute the phase transition of Er_0.1_Yb_0.9_Fe_2_O_4_ around 300 K to a 3D-2D CO phase transition with increasing temperature, as marked by the 1372 and 1554 cm^−1^ peaks. Quantitatively, these Raman shift results provides explicit energy band information that the 2D CO is 170 meV and 193 meV higher that the 3D CO, respectively. Such an energy gap (Fig. 4c) exists at 300 ~ 400 K.

### Double 2D CO states with THz energy

In Fig. 2, the Raman peaks emerge at the temperature above 300 K are attributed to the 3D-2D phase transition. In the 2D CO state the atoms are closer than those in the 3D CO state, so the energy of the 2D CO is higher. Significantly we have observed two separate peaks, which indicated that the 2D CO is indeed composed of non-degenerate double states. The energies are 170 meV (41.2 THz) and 193 meV (46.6 THz) higher than that of the 3D CO state, respectively (Fig. 4c). We speculate that these non-degenerate double 2D CO states might come from two separate and different charge orderings within the W-layer: one comes from the nearest neighbors and the other comes from the next-to-nearest neighbors, thus forming charge orderings along different directions of the crystal lattice. Since collective CO or SO states are very sensitive to distances between atoms, this might explain the non-degeneracy of the double THz states. If this is true, then bi-directional ferroelectricity can be expected, which must co-exist together (although not all the experimental means are sensitive to it).

Alternatively, these double 2D CO states might originate from a predicted fact^9^ that the W-layer of the iron ions are asymmetric and composes of a Fe^2+^-rich layer and a Fe^3+^-rich layer simultaneously. Thus double long-range 2D CO develop within each sub-layer plane, but with non-degenerate energies. If the latter scenario is true, then our result provides direct experimental proof of the theoretical prediction of the bifurcated charge distribution and ordering within the W-layer in Ref. 9.

There is also a third possibility that this double CO state is originated from the doping of Yb atoms. The CO state reflects the properties of the Iron ions of the W layer, whereas the doping of Er/Yb ions does not affect the charges of Iron ions directly. However, from Fig. 2 and 3, for the temperature range accommodating the double CO states (Fig. 2 & 3), it can be seen that all the phonon modes (see below for discussion) show variations (in both frequency and intensity) with temperature, leading us to claim that the emergence of the double THz 2D CO is correlated to a structural softening. Note that both the phonon softening and the 2D CO states are associated to the varying lattice constants with temperature. The effect of doping might be conveyed by charge-lattice interactions. If this is true, then what we have found is a realization of generating double CO states by using chemical doping.

The observation of the double state of CO is unlikely due to slightly different states possibly due to the polycrystalline multi-domains, because we did not observe other multiple peaks and the two Raman peaks are clearly separated. In any case, further investigation is needed to confirm the origin of the double CO states.

### Spin Ordering State

Among the various investigations of the magnetic properties of RFe_2_O_4_, neutron scattering is most extensively used, with the initial identification of a ferrimagnetic ordering below 240 K in LuFe_2_O_4_^31^. Further neutron investigation with single crystal demonstrated that the ferrimagnetic spin ordering is of 3D nature^22^. Then the results of X-ray absorption spectroscopy suggested that in the W layer the Fe^2+^ ions all align ferromagnetically and the Fe^3+^ ions experience triangular frustration, emerging two possible configurations each preserving a 1:2 ferrimagnetic ordering^20^. So far whether the two configurations are degenerate is not clear and no further evidence has been found to further identify the spin ordering thus formed. Particularly, no Raman scattering or other optical spectroscopy methods have been reported on observing such a spin ordering. Here in our result, when *T* is below 210 K, a new mode was unambiguously observed (mode 4 in Figs. 1–3), with a characteristic energy of 45 meV (10.7 THz). Comparing with the available neutron and X-ray data we attribute this mode to the ferrimagnetic spin ordering of the system. Such a ground state of the spin structure is of long-range ordering nature and is usually elusive in a typical Raman scattering with linearly polarized probe to the center of the Brillion zone^32^,^33^,^34^. However, we have a polycrystalline sample immersed in the scatterings and breakings of the symmetry, which greatly relaxes the dipole transition restriction. In Fig. 4a–b, we show the suggested 3D spin ordering configuration of the system at temperatures below 210 K^20^. The fact that we have observed only one such spin ordering state implies that the two suggested configurations are either degenerate or non-degenerate with very small energy difference. From Fig. 3 it can be seen that this spin ordering state might be associated with the E_g_^3^ mode phonon, where the interaction between them greatly modifies the phonon mode energy, especially at the low temperature regime.

### Raman- and IR-active phonon modes

The RFe_2_O_4_ family crystals share a layered rhombohedral lattice structure (with space group 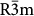) at room temperature. It consists of alternating Fe-O double-layers (W layer) and R-O layers (U layer) stacked along the *z*-axis. In the W layer two sheets of triangular corner-sharing FeO_5_ trigonal bipyramids constitute a layer of hexagonal Fe_2_O_2.5_, where the two sheets are shifted by 1/3 *x* in the *xy* plane^2^. In the U layer the R_2_O_3_ atoms form a single flat layer instead. Such a RFe_2_O_4_ crystal structure preserves the center inversion symmetry. The lattice vibrations in RFe_2_O_4_ is constituted of six Raman-active modes and six infrared (IR)-active modes, *i.e.*, 3A_1g_ + 3E_g_ for Raman and 3A_2u_ + 3E_u_ for IR^5^,^6^. The displacement symmetry of the A_1g_ mode is along the *c*-axis, whereby the R-Fe atoms vibrate in opposite directions along the *c*-axis. The displacement symmetry of the E_g_ mode is along the *xy* plane, whereby the atoms vibrate in opposite directions along to the R-O and Fe-O bonds, respectively. Er_0.1_Yb_0.9_Fe_2_O_4_ has been shown to have a similar structure to that of LuFe_2_O_4_ via X-ray experiment^7^.

In conclusion, we have investigated the orderings and phase transition in the polycrystalline Er_0.1_Yb_0.9_Fe_2_O_4_ using Raman scattering. We for the first time found the double 2D CO states in the system and directly obtained the energy gap between the 2D and 3D CO states, which is at the THz range. Furthermore, a ferrimagnetic spin ordering mode was also observed, which has not been reported before in Raman scattering. Meanwhile two IR-active modes were also observed due to the symmetry breaking at the domain boundaries. Our investigation adds new feature to the fine structure of RFe_2_O_4_ system, which shines light to further investigations of other layered quantum materials.

## Methods

### Sample preparation

Our sample was synthesized using a conventional solid state reaction method. The details of the growth are described in Ref. 7. In addition, the sample surface was carefully polished mechanically before the Raman measurement. Due to the polycrystalline nature of the sample, the surface displays irregular dents. We measured only at the smooth part of the sample surface.

### Variable-temperature Raman spectroscopy

All Raman data shown in this paper were obtained using a near-field con-focal spectrometer. The sample was kept in a continuous flow liquid nitrogen cryostat. The cryostat contains a heating element, making it possible to operate at temperatures ranging from 78 to 403 K. Temperature fluctuation was kept as low as 0.1 K. A 532 nm linearly polarized continuous wave laser beam with a power of 0.9 mW was used. An objective lens with 50× magnification was used to focus the laser light onto a 100 µm slit of the spectrometer which has a spectral resolution of 2 cm^-1^. X-ray diffraction measurements were carried out to characterize the crystal lattice structure and phase purity. No impurity phase was observed, and the lattice constants are described in Ref. 7.

## Figures and Tables

**Table 1:** Observed active modes in Er_0.1_Yb_0.9_Fe_2_O_4_ (at room temperature, if not specified)

Mode Number	1	2	3	4	5	6
Frequency (cm^−1^)	129	216	274	360(78 K)	412	463
Symmetry (Mode type)	E_u_^1^ (IR)	E_g_^1^ (Raman)	A_1g_^1^ (Raman)	(SO)	A_1g_^2^ (Raman)	E_g_^2^ (Raman)

Mode Number	7	8	9	10	11
Frequency (cm^−1^)	554	576	686	1372	1554
Symmetry (Mode type)	E_u_^3^ (IR)	E_g_^3^ (Raman)	A_1g_^3^ (Raman)	(CO1)	(CO2)
